# Effect of Different Egg Products on Lipid Oxidation of Biscuits

**DOI:** 10.3390/foods9111714

**Published:** 2020-11-22

**Authors:** Vito Verardo, Maria Cristina Messia, Emanuele Marconi, Maria Fiorenza Caboni

**Affiliations:** 1 Department of Nutrition and Food Science, University of Granada, Campus of Cartuja, 18071 Granada, Spain; 2 Institute of Nutrition and Food Technology ‘José Mataix’, Biomedical Research Center, University of Granada, Avda del Conocimiento sn., Armilla, 18100 Granada, Spain; 3 Dipartimento Agricoltura, Ambiente e Alimenti, Università degli Studi del Molise, via F. De Sanctis, I-86100 Campobasso (CB), Italy; messia@unimol.it (M.C.M.); marconi@unimol.it (E.M.); 4 Department of Agricultural and Food Sciences, University of Bologna, piazza Goidanich 60, 47521 Cesena (FC), Italy; maria.caboni@unibo.it; 5 Inter-Departmental Centre for Agri-Food Industrial Research (CIRI Agroalimentare), University of Bologna, via Quinto Bucci 336, 47521 Cesena (FC), Italy

**Keywords:** eggs, biscuits, cholesterol oxides (COPs), peroxide value (PV), oxidized fatty acids (OFA)

## Abstract

Egg products are one of the main ingredients used in bakery industries, and they contain cholesterol. Cholesterol suffers several chemical changes during the food processes, allowing some potentially toxic compounds called cholesterol oxidized products (COPs). Thus, the aim of this work was to study the evolution of lipid oxidation from eggs to egg products, and to evaluate the influence of egg products on COPs formation in biscuits formulated with them. The results confirmed that spray-drying technology improves the cholesterol oxidation 2.6 times compared to pasteurized eggs. Biscuit samples showed a COPs content that is strictly related to the egg products used. Samples formulated with spray-dried eggs noticed lower amounts of COPs compared to those formulated with pasteurized eggs. It is important to stress that COPs composition was different between the two samples, underlining that the kinetic of COPs formation is dependent on the type of egg products.

## 1. Introduction

Bakery products are a kind of food that is eaten during breakfast or as snacking [1]. Among others, biscuits are one of the most consumed bakeries. Their formulation includes several ingredients such as flour, fat, sugars, and sometimes milk powder or egg products have also been added [1,2]. The ingredients used during formulation and the processes, including the packaging and storage, are responsible for the texture, sensorial characteristics, stability and shelf life of these products [3]. Besides, biscuits are usually formulated with saturated fats; the addition of other ingredients could alter their stability [1,3]. In fact, ingredients of animal origin that are used during biscuit formulation (i.e., butter, egg products, etc.) are a source of cholesterol; during the food processes, this compound produces its oxidized derivatives that are known as COPs (cholesterol oxidation products) [4]. COPs are more polar than cholesterol thanks to their functional groups that are hydroxyl, keto, hydroperoxy, epoxy, or carboxyl moieties [5]. It has been noticed that these compounds are adsorbed and play an important role in the modulation of cholesterol/lipid metabolism and membrane functionality and in the chronic inflammation [5]. Their formation in foods is principally related to the lipid oxidation reactions caused by heating treatments; however, other important factors such as the presence of oxygen, light exposure, etc., are also involved in their formation [6]. Generally, the mechanisms of formation of COPs change if thermo-oxidation or photo-oxidation is considered [7]. Thermo-oxidation occurs after the formation of reactive oxygen species; heating processes accelerate the production of peroxy radicals via hydroperoxyde homolysis [7], and after that, several reactions evolve producing different oxysterols [8]. The presence of photosensitizers and light causes the excitation of triplet oxygen to singlet oxygen that has a strong reactivity with the cholesterol double bond, generating the oxysterols without a radical mechanism and induction period [9].

COPs are described in foods containing egg products [10] and other animal products [11]; the most abundant COPS are 7-ketocholesterol, epoxide and hydrocholesterol derivatives [8,9,10,11]. To limit the COPs formation, several strategies including the use of antioxidants have been reported. To this aim, synthetic and natural antioxidants have been used [12]. However, the use of natural antioxidants recovered by food by-products could represent a good opportunity in terms of the sustainable food industry [13]. This approach was successfully applied to egg [14,15,16] and other animal food industries [17].

The presence of COPs in animal products, and particularly in egg products, is widely described; however, the data on their presence in biscuits containing egg products are scarce. Thus, the aim of this work was to evaluate the influence of different egg products on the lipid oxidation of biscuits. To reach this goal, firstly, the influence of the thermal processes (pasteurization and spray-drying) for the production of egg products on lipid stability was evaluated. Finally, the produced egg products were used as biscuit ingredients. Thus, lipid oxidation in the biscuits formulated with pasteurized and spray-dried eggs was also determined.

## 2. Materials and Methods

### 2.1. Chemicals

Solvents and reagents were analytical grade and, unless specified, were purchased from VWR (Darmstadt, Germany). The analytical standards used for identification of COPs (7-ketocholesterol (7-KC), 7-α-hydroxycholesterol (7-α-HC), 7-β-hydroxycholesterol (7-β-HC), α-epoxycholesterol (α-CE), β-epoxycholesterol (β-CE) and 19-hydroxycholesterol (19-HC)) were supplied by Steraloids (Wilton, NH, USA). Cholesterol was purchased from Sigma Aldrich (St. Louis, MO, USA).

### 2.2. Samples

Fresh eggs (FE), pasteurized eggs (PE) were furnished by a local factory. Spray-dried eggs (SPE) were produced from the same pasteurized eggs using a mini spray dryer Büchi B-290 (Buchi Labortechnik AG, Flawil, Switzerland), located at University of Molise. The drying medium was air, and its flow was increased or decreased regulating the aspirator speed. The process was performed at the optimized parameters of: inlet air temperature of 100 °C, outlet temperature of 57 °C, aspiration 100% and feed flow rate of 4 mL/min. After the spray-drying, the drying yield was of about 92%, determined as the % of the powder weight collected from the receiver to the amount initial of solids contained in the feed solution. At the end of the process, the spray-dried eggs were sealed in plastic bag and stored at +4 °C until use.

Biscuits were produced by means of a bakery pilot plant located at the University of Molise. The ingredients used for the formulation were reported in Table 1.

The ingredients were mixed in a spiral kneader and then were kneaded for 30 min. The dough was laminated and biscuits were formed before cooking (180 °C for 15 min) in a rotary oven.

Three batches of biscuits were done using the same conditions.

### 2.3. Moisture Determination

To determine the moisture of samples, the ICC method 110/1 [18] was used. Thus, 7 g of grounded biscuits were dried in an oven for 90 min at 130 °C until constant weight.

### 2.4. Ash Content

Ash content was determined according to the ICC method 104/1 [19]; 1 g of grounded biscuits was collected in a porcelain crucible and put in muffle at 525 °C until the residue was white or nearly white.

### 2.5. Lipid Extraction for Fat Composition

Soxhlet method AOAC 14.088-14.089 [20] was employed to evaluate the lipid content. Briefly, 6 g of grounded biscuit were extracted in a Soxtec apparatus (1045 Soxtec Extraction Unit, Foss Tecator).

### 2.6. Protein Determination

The Dumas combustion method, AOAC method 992.23 (AOAC, 2000) was applied to determine the protein content (Nx6.25) using a Leco nitrogen determiner, model FP 528 (Leco Corp., St. Joseph, MI, USA). Pasteurized liquid egg was weighed (250 mg) in a tin capsule (Leco tin capsule 502-040); the powdered egg samples were weighed (100 mg) in tin foil (Leco tinfoil cups 502-186), using a foil holder (Leco 604-493) and twisting the ends of the foil to form a teardrop-shaped pocket.

### 2.7. Lipid Extraction for Lipid Oxidation Analysis

Samples were ground with an Ika-Werke food processor (Staufen, Germany). The lipid fraction of the egg products (3 g) and biscuit samples (15 g) was extracted using the Folch’s procedure [10]. Each sample was extracted three times (*n* = 3).

### 2.8. Peroxide Determination

The International Dairy Federation method of Shantha and Decker [21] was used to determine the peroxide values (PV). Then, 0.05 g of fat was added by a Fe (II) and ammonium thiocyanate solutions. The intensity of a red-violet complex of strong absorption at 500 nm was evaluated.

### 2.9. Cholesterol and COPs Determination

Cholesterol and cholesterol oxides (COPs) were extracted after a cold saponification [22] at room temperature. Briefly, 250 mg of lipids were added, of 25 µL of 19-hydroxycholesterol (0.5 mg/mL) and 500 μL of dihydrocholesterol (2.02 mg/mL). A tenth part of unsaponifiable matter was used for cholesterol quantitation (using the dihydrocholesterol as standard). The rest of the unsaponifiable matter (9/10) was purified on NH2 solid-phase extraction according to Rose-Sallin et al. [23], in order to obtain the COPs fraction (using the 19-hydroxy-cholesterol as standard). After silylation [24], the extracts were dried under gentle nitrogen flow, dissolved in 100 µL hexane and used for GC analyses. The cholesterol and COPs were separated on a ZB-5 (30 m × 0.25 mm i.d., low bleeding for MS) from Phenomenex (Torrance, CA, USA) coated with a 0.25 µm film of 5% phenylpolysiloxane and of 95% dimethylpolysiloxane column. A GC-FID Clarus 500 Perkin Elmer instrument (Waltham, MA, USA) was used for quantitation scope; the GCMS-QP2010 Plus (Shimadzu Corp., Tokyo, Japan) was employed to identify the COPs. Identification was carried out comparing the retention times and the mass spectra obtained in the samples and those of pure standards.

### 2.10. Oxidized Fatty Acids Analysis (OFA)

The OFA accumulation was evaluated according to Rovellini and Cortesi [25]. Briefly, 20 mg of fat was methylated with diazomethane and subsequently benzylated. Oxidize fatty acids were determined by HPLC analysis using an Agilent 1200 system (Agilent technologies, Santa Clara, CA, USA) equipped by a diode array detector. The OFA were separated on a Luna C18 column of Phenomenex (Torrance, CA, USA), 250 × 4.6 mm (i.d.), 5 μm.

### 2.11. Statistical Analysis

The results reported in this work are the averages of three repetitions (*n* = 3). Pearson’s linear correlations and Tukey’s honest significant differences (one-way ANOVA) (at the *p* < 0.05 level) were calculated with Statistica 6.0 software (2001, StatSoft, Tulsa, OK, USA).

## 3. Results and Discussion

### 3.1. Chemical Composition and Oxidative Status of Egg Products

The chemical composition of egg products was assessed to evaluate differences induced by different treatments on them (Table 2). As expected, fresh and pasteurized eggs were characterized by high moisture values (76.4% and 73.8%, respectively). The optimized parameter used for spray-drying allowed to produce an egg powder with low moisture content (2.3%), high protein and lipid contents (48.8% d.w. and 48.0% d.w., respectively) and low water activity (a_w_) value (0.252 ± 0.02).

Moisture content and water activity are important prerequisites for storage stability of spray-dried powders with good handling characteristics such as high flow ability, low stickiness and agglomeration [26]. Instead, a_w_ is a key marker for spray-dried powder, because it can strongly influence the shelf life of the powder produced.

**Figure 1:**
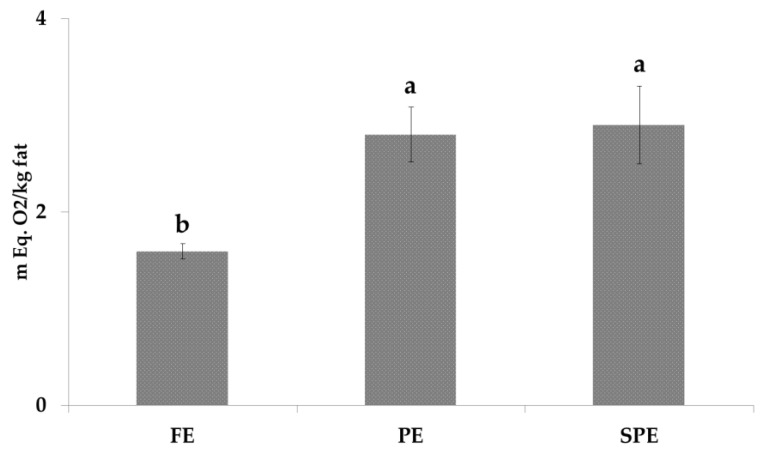
PV amounts (mEq. O2/kg fat) in fresh eggs (FE), pasteurized (PE) and spray-dried (SPE) egg products. Different letter means statistical difference (*p* < 0.05). PV: peroxide values.

Peroxide value (PV) was determined in order to establish the influence of thermal process on primary lipid oxidation products. As reported in Figure 1, PV increased from fresh to spray-dried products showing 1.59, 2.80 and 2.9 mEq. O_2_/kg of fat for FE, PE and SPE, respectively. The increment was 1.8 times higher after pasteurization. A different trend was noticed in a previous work [27], where the PV value of spray-dried eggs reached 4.4 mEq. O_2_/kg of fat. It is assumed that the different conditions used for the spray-drying treatment applied in this trial (lab scale instead of industrial scale) have positively affected the PV value inducing a lower oxidation of the eggs.

**Figure 2:**
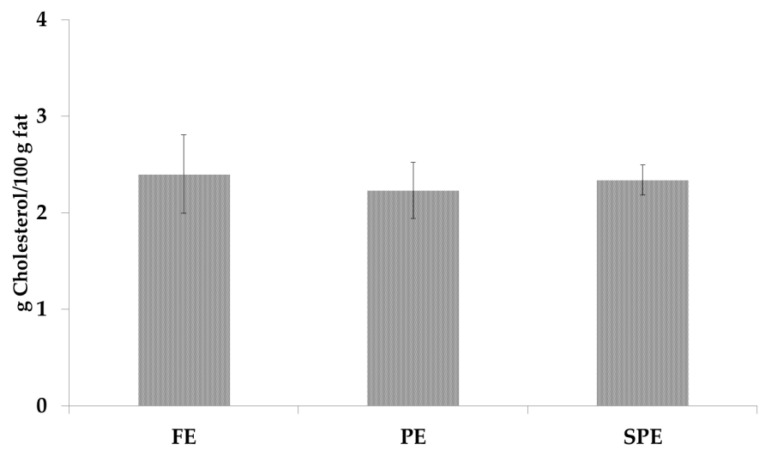
Cholesterol content in fresh eggs (FE), pasteurized eggs (PE) and spray-dried (SPE) egg products.

Unsaponifiable extracts, before the purification by solid phase extraction, was used to determine the cholesterol content (Figure 2).

Mean of cholesterol content was 2.3 ± 0.1 g/100 g of fat; similar content was previously obtained by Caboni et al. [28], and any statistical difference (*p* < 0.05) was reported among the three samples.

**Figure 3:**
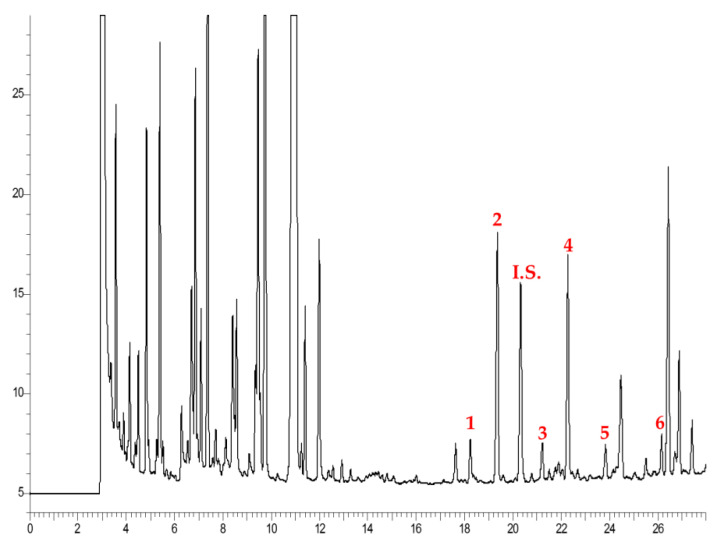
GC–FID chromatogram of COPs in biscuits.

The COPs contained in the different egg products were determined by GC–FID (Figure 3), and their identification was confirmed by co-elution with commercial standards and by GC–MS.

Five COPs were identified; briefly, compounds 1 and 3 reported a base peak at 456 m/z typical of 7α-HC and 7β-HC. Peaks 4 and 5 showed two major peaks at 474 and 384 m/z, thus they were assigned to β-CE and α-CE, respectively. Finally, peak 6 with m/z 472 was identified as 7-KC [29]. Peak 2 is related to cholesterol that was not totally eliminated during the solid-phase extraction step. Table 3 reported the COPs content in the different egg products.

As noticed in the literature [30], the data obtained in this work confirmed that the spray-dried sample reported the highest COPs content that increased 2.6 times compared to pasteurized egg product. Consequently, the percentage of cholesterol affected by the oxidation in SPE was about 3 times higher than the PE sample. As expected, 7-KC was the first oxidized compound amounting from 26.2% of total COPs in PE sample. This compound is formed from dehydration of cholesterol hydroperoxides produced in the initial heating period, and after the dehydrogenation of 7-HC [31]. The formation of 7-KC occurred during the first stages of the oxidation; because of that it was usually used as a marker of cholesterol oxidation [32]. However, SPE samples showed 7-HC isomers as the main COPs. In fact, the sum of 7-α-HC and 7-β-HC was 30.6 and 55.9% of total COPs in PE and SPE sample, respectively; thus, the sum of 7-KC and the two 7-HCs is 56.8 and 68.5% of total COPs in PE and SPE, respectively. These results confirmed that 7α-OOH and 7β-OOH that could be detected at the beginning of the thermal process were reduced during heating to 7-oxigenated products that are more stable products [4,7,8].

### 3.2. Chemical Composition of Biscuits

The chemical composition of biscuits formulated with pasteurized eggs (BPE) and with spray-dried pasteurized eggs (BSPE) was also determined (Table 4). No statistical differences (*p* < 0.05) are noticed between BPE and BSPE samples which showed average moisture, protein, lipid and ash contents equal to 8.3%, 0.77%, 7.8% and 18.4%, respectively; this was confirmed, as the same amounts (based on dry weight) of egg products were enclosed in the formulation of the two biscuit samples. Fat composition includes about 56% of unsaturated fatty acids (mainly oleic and linoleic acids) and 44% of saturated fatty acids (mainly palmitic and stearic acids).

### 3.3. Lipid Oxidation in Biscuits

**Figure 4:**
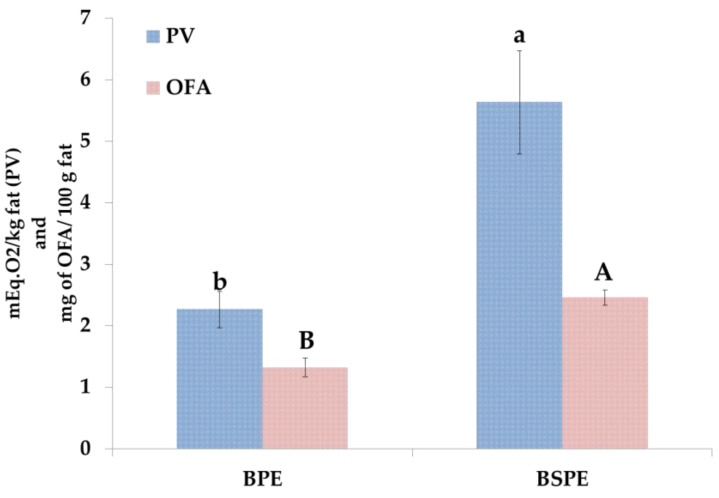
PV and OFA content in biscuits. Different letter means statistical difference (*p* < 0.05). PV: peroxide value; OFA: oxidize fatty acids.

To determine the lipid oxidation of BPE and BSPE samples, peroxide value (PV), oxidize fatty acids (OFA) and COPs were analyzed. Figure 4 shows the PV and OFA values in biscuit samples.

PV is usually used as oxidation index of the primary oxidation stage. This value was obtained quantifying the peroxides by calibration curve of Fe (III) (y = 0.0271x + 0.0652, r^2^ = 0.9994). BPE showed the lowest value of peroxides that was 2.4 times lower than BSPE. These data confirmed that higher oxidation state was obtained when high oxidized raw ingredients were used in the recipe. In fact, SPE egg product is more oxidized than PE, and this was also reflected in the biscuits formulated with these ingredients. However, both samples reported PV lower than 10 mEq. O_2_/kg of fat, that was reported as the highest limit before considering these kinds of products sensorially unacceptable by the consumers [33]. The PV obtained is in the same order of magnitude of those obtained by other authors [33,34]. Oxidized fatty acids (OFA) noticed the same trend of PV; BPE values were about two times lower than BSPE, confirming the higher oxidation state of the samples formulated with SPE ingredient. Similar values were determined by Verardo et al. [35] in biscuits. A positive correlation was found between PV and OFA (r = 0.9039 *p* < 0.05).

### 3.4. Cholesterol and COPs Content in Biscuits

**Figure 5:**
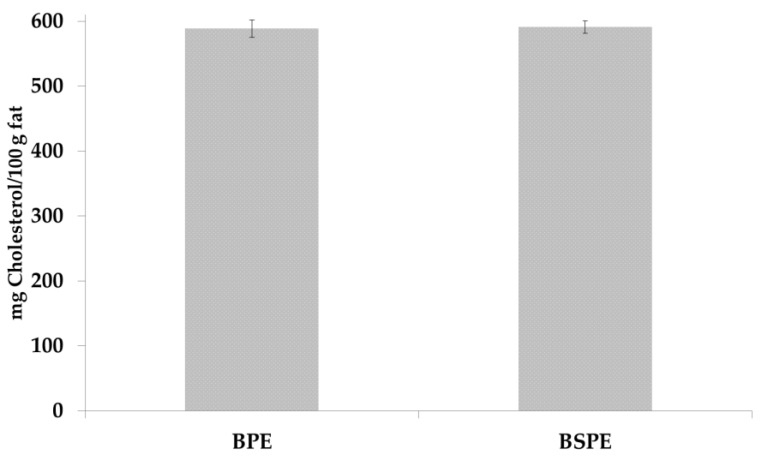
Cholesterol content in biscuits formulated with pasteurized (BPE) and spray-dried (BSPE) egg products.

Figure 5 reported the cholesterol content in biscuit samples. BPE and BSPE contained 588.7 and 590.8 mg of cholesterol/100 g of fat; these values agreed with the amounts of cholesterol reported in biscuits by Lercker and Rodriguez-Estrada [32].

No statistical differences were noticed among the cholesterol content in the formulated biscuits, confirming the similar formulation between the samples.

Table 5 shows the COPs content in biscuits. A statistical difference (*p* < 0.05) was noticed between BPE and BSPE samples; surprisingly, biscuits formulated with pasteurized egg products reached the highest value (23.7 vs. 20.4 µg/g fat).

The total COPs content in the formulated samples is in the same order of magnitude as those obtained in other foods such as chicken breast, hake fillets, chicharrón, machaca, sardines, shrimp, cheese [4,9], but higher than in others [4,6,9].

Statistical differences (*p* < 0.05) have been found about the single COPs between biscuit samples. 7-KC decreased of 34% in BSPE compared to BPE. As previously reported, this compound is formed in the first stage of oxidation [32]; however, egg cholesterol in the BSPE sample suffered three thermal treatments as pasteurization, spray-drying and the oven-cooking. According to literature data [5,36], 7-KC thermal stability is low if compared with other COPs, thus its degradation during these thermal treatments could be occurred, forming volatile compounds [29] and/or involving 7-KC in the formation of Maillard reaction products [37]. Among other COPS, also 7-β-HC decreased from BPE to BSPE sample (−27.7%); according to Min et al. [36], this compound decreases during heating treatments.

Contrary to the other COPS, cholesterol epoxi-derivatives increased from BPE to BSPE. The β-CE epimer was the most abundant because this conformational isomersim is favored [29]; however, both epimers are higher in BSPE than BPE. The cholesterol epoxides were produced from the reaction of 7-hydroperoxides with cholesterol [4,7,8,31] with a second-order kinetic type. Thus, the results reported in our samples underlined that the kinetic of COPs formation is strictly dependent on the grade of oxidation of the raw material used to produce the biscuits. Briefly, the presence of hydroperoxides is the start point necessary for epoxides formation [5,8,31]. Epoxides derivatives represented the 30% of total COPs in BSPE, instead they are only 12.8% of the total COPs in BPE. Positive correlations were found between sum of epoxides and PV (r = 0.9861, *p* < 0.05) and between sum of epoxides and OFA (r = 0.9855, *p* < 0.05); this correlation was highlighted as the high presence of oxidation products in SPE ingredient that favored the formation of epoxides during BSPE production. Moreover, negative correlations were reported between sum of epoxides and sum of 7-HC (r = −0.9630, *p* < 0.05) and between sum of epoxides and 7-KC (r = −0.9612, *p* < 0.05).

## 4. Conclusions

The results obtained in this work confirmed that thermal processes such as pasteurization and spray-drying enhanced the lipid oxidation of egg products. It is important to stress that spray-drying carried out at lab scale produces lower amounts of COPs compared to the industrial scale (data obtained in previous works).

After baking, the samples formulated with pasteurized eggs reported higher COPs content compared to those containing pasteurized/spray-dried eggs; however, their composition was different. Both samples showed 7-β-HC and 7-KC as main COPs, but samples formulated with spray-dried eggs reported also higher amounts of β-epoxide isomers, confirming that a second-order kinetic type is favored in these kinds of samples. This could be due to the higher amounts of peroxides in biscuits containing spray-dried egg products. To our knowledge, this is the first time that the influence of type of egg products on the COPs formation has been reported in biscuits. However, further analyses will be done in order to study other oxidation products that could be originated from the cholesterol oxidation and their evolution during the shelf life.

## Figures and Tables

**Table 1:** Recipes of biscuits formulated with PE and SPE.

Ingredients	Biscuit with PE (BPE)	Biscuit with SPE (BSPE)
Refined wheat flour (g)	2000	2000
Butter (g)	600	600
Sucrose (g)	600	600
Milk (g)	100	100
Water (g)	0	300
Egg product (g)	575	150
Ammonium bicarbonate (g)	30	30

PE, pasteurized eggs; SPE, spray dried pasteurized eggs.

**Table 2:** Chemical composition (g/100 g) of egg products.

Sample	Moisture (f.w.)	Protein (d.w.)	Lipid (d.w.)	Ash (d.w.)
**FE**	76.4 ± 0.15	51.3 ± 2.78	44.9 ± 2.80	3.86 ± 0.02
**PE**	73.8 ± 0.01	48.5 ± 2.14	45.0 ± 3.32	4.20 ± 0.09
**SPE**	2.3 ± 0.01	48.8 ± 0.19	48.0 ± 0.20	3.65 ± 0.14

FE, fresh eggs; PE, pasteurized eggs; SPE, spray dried pasteurized eggs.

**Table 3:** COPs content (µg/g fat) in FE, and PE and SPE egg products.

COPs	FE	PE	SPE
7-α-HC	<LOD	6.68 ± 0.09 b	20.67 ± 0.91 a
7-β-HC	<LOD	1.85 ± 0.09 b	19.18 ± 0.43 a
β-CE	<LOD	7.44 ± 0.45 b	15.04 ± 0.28 a
α-CE	<LOD	4.62 ± 0.19 b	7.38 ± 0.22 a
7-KC	<LOD	7.30 ± 0.46 b	9.03 ± 0.31 a
Sum	<LOD	27.89 ± 0.30 b	71.29 ± 0.86 a
% cholesterol affected by oxidation	-	0.12	0.30

<LOD (limit of detection) = 0.10 mg/g; different letters in the same line mean statistical differences (*p* < 0.05).

**Table 4:** Chemical composition of biscuits (g/100 g f.w.).

Sample	Moisture	Protein	Lipid	Ash	Carbohydrates *
**BPE**	8.1 ± 0.05 a	7.7 ± 0.02 a	18.3 ± 0.10 a	0.74 ± 0.02 a	65.2
**BSPE**	8.5 ± 0.01 a	7.8 ± 0.00 a	18.4 ± 0.15 a	0.80 ± 0.07 a	64.5

* Calculated by difference. Different letters in a column indicate significant differences (*p* < 0.05).

**Table 5:** COPs content (µg/g fat) BPE and BSPE egg products.

COPs	BPE	BSPE
7-α-HC	3.77 ± 0.46 a	2.56 ± 0.55 a
7-β-HC	9.08 ± 0.30 b	6.56 ± 0.37 a
β-CE	2.02 ± 0.25 b	4.59 ± 0.39 a
α-CE	1.00 ± 0.03 b	1.57 ± 0.13 a
7-KC	7.82 ± 0.13 b	5.17 ± 0.23 a
Sum	23.69 ± 1.11 b	20.44 ± 0.16 a
% cholesterol affected by oxidation	0.40	0.35

Different letters in the same line mean statistical differences (*p* < 0.05).
